# Tree-Based Unrooted Phylogenetic Networks

**DOI:** 10.1007/s11538-017-0381-3

**Published:** 2017-12-13

**Authors:** A. Francis, K. T. Huber, V. Moulton

**Affiliations:** 1 Centre for Research in Mathematics, Western Sydney University, Sydney, Australia; 2 School of Computing Sciences, University of East Anglia, Norwich, UK

**Keywords:** Phylogenetic tree, Phylogenetic network, Tree-based network, Hamiltonian path

## Abstract

Phylogenetic networks are a generalization of phylogenetic trees that are used to represent non-tree-like evolutionary histories that arise in organisms such as plants and bacteria, or uncertainty in evolutionary histories. An *unrooted* phylogenetic network on a non-empty, finite set *X* of taxa, or *network*, is a connected, simple graph in which every vertex has degree 1 or 3 and whose leaf set is *X*. It is called a *phylogenetic tree* if the underlying graph is a tree. In this paper we consider properties of *tree-based networks*, that is, networks that can be constructed by adding edges into a phylogenetic tree. We show that although they have some properties in common with their rooted analogues which have recently drawn much attention in the literature, they have some striking differences in terms of both their structural and computational properties. We expect that our results could eventually have applications to, for example, detecting horizontal gene transfer or hybridization which are important factors in the evolution of many organisms.

## Introduction

Let *X* be a finite set with $|X| \ge 1$. An *unrooted phylogenetic network N (on X)* (or *network N (on X)* for short) is a connected, simple graph (*V*, *E*) with $X \subseteq V$, every vertex has degree 1 or 3, and the set of degree 1 vertices (or *leaves*) is precisely *X*. A *phylogenetic tree* on *X* is a network which is also a tree. Phylogenetic trees and networks are commonly used by biologists to represent the evolution of species; in this setting the set *X* usually denotes a collection of species. Networks have interesting mathematical and computational properties (see, e.g. Huber et al. 2016; Gambette et al. 2012; Semple and Steel 2006), and they can be generated from biological data using software packages such as T-REX (Makarenkov 2001) and Splitstree (Huson and Bryant 2006). In addition, networks have been used to study the genome fusion origin of eukaryotes (Rivera and Lake 2004) and as a tool in biogeography studies (Legendre and Makarenkov 2002).

**Fig. 1:**
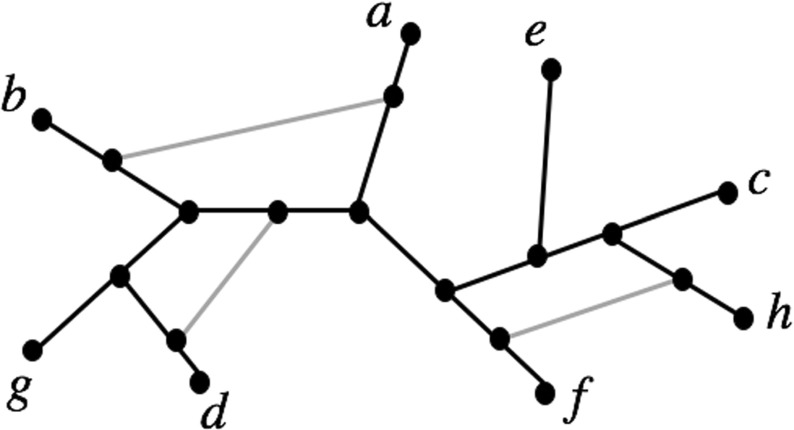
A tree-based network that has been constructed from a phylogenetic tree with leaf set $\{a,b,\dots ,g\}$ by adding in 3 edges (in grey). Note that the tree is also a spanning tree for the network

The T-REX software constructs networks (also called reticulograms) by adding edges into a phylogenetic tree (Makarenkov 2001) (see, e.g. Fig. 1). Using this approach, many different networks can be constructed from starting with the collection of all phylogenetic trees. However, it is not possible to construct every possible network in this manner (see, for example, Fig. 4 below). Indeed, the networks that can be constructed in this way are of precisely the following type (cf. Francis et al. 2018).

### Definition 1

A network is *tree-based (on X)* if there is a spanning tree^1^ in *N* whose leaf set is equal to *X*.

Note that in the following we call any spanning tree in *N* with leaf set *X* a *support tree (for N)*.

Recently, a great deal of interest has been generated concerning rooted tree-based networks. These are leaf-labelled networks whose underlying graph is a directed acyclic graph with a single root which can be constructed from a rooted phylogenetic tree by adding in extra arcs (see Sect. 5 for precise definitions). In particular, rooted tree-based networks were introduced in (Francis and Steel 2015) and their structural properties have been studied in Francis et al. (2018), Hayamizu (2016), Jetten and van Iersel (2016), Semple (2016), Zhang (2016). In addition, various computational properties of these networks have been considered (Anaya et al. 2016; Francis and Steel 2015).

Biologically, tree-based networks are a natural object of interest because their presentation of reticulations reflects assumptions about the underlying biological events (such as horizontal gene transfer or hybridization). Such events are regarded as occurring between taxonomic units (such as species) that also undergo vertical evolution; hence they are thought of as arcs between “tree” arcs. Biologists want to detect and understand horizontal evolution through events such as gene transfer and hybridization since this process plays an important role in the evolution of many organisms (e.g. in bacteria and plants). Moreover, detecting horizontal evolution can be useful in applications, e.g. horizontal gene transfer is the primary mechanism for the spread of antibiotic resistance in bacteria (Gyles and Boerlin 2014).

In this paper, we introduce tree-based networks in an unrooted setting and present various results concerning them. Understanding such networks in an unrooted context has similar value to the study of phylogenetic trees in the unrooted context: we are able to allow for uncertainty in the placement of the root. As we shall see, although tree-based networks have certain properties in common with their rooted analogues, they can behave quite differently both in terms of their structural and computational properties.

The outline of the paper is as follows. In the next section, we introduce some relevant basic terminology and also present a way to decompose a network into simpler, easier to understand pieces. In Sect. 3, we consider the computational problem of deciding whether or not a given network *N* is tree-based. For this we introduce two novel decision problems and contrast our findings with the analogous situation for rooted phylogenetic networks. In Sect. 4, we show that for every non-empty set *X* there exists a tree-based network *N* on *X* such that every phylogenetic tree *T* on *X* is a base-tree for *N*, and we also establish an explicit relationship between rooted and unrooted tree-based networks (Theorem 4). In Sect. 5, we characterize level-1 networks in terms of tree-based networks. We conclude with Sect. 6 where we outline research directions that might be of interest.

## Decomposing Tree-Based Networks

We begin by showing that networks can be decomposed into simpler pieces, which can then be used to deduce properties of the full network. Note that decomposition results have also been proven for rooted tree-based networks, although these are quite different in nature (see, e.g. Zhang 2016).

We begin by presenting some definitions. A *cut-edge*, or *bridge*, of a network is an edge whose removal disconnects the graph. A cut-edge is *trivial* if one of the connected components induced by deleting the cut-edge is a vertex (which must necessarily be a leaf). A *simple* network is one all of whose cut-edges are trivial (so for instance, note that trees on more than 3 leaves are *not* simple networks). A *blob* in a network is a maximal subgraph that has no cut-edge and that is not a vertex (Gambette et al. 2012). For example, the network in Fig. 1 contains one non-trivial cut-edge and two blobs.

Now, given a network *N* and a blob *B* in *N*, we define a simple network $B_N$ by taking the union of *B* and all cut-edges in *N* incident with *B* (the leaf set of $B_N$ is just the set of end vertices of these cut-edges that are not already a vertex in *B*).

### Proposition 1

Suppose *N* is a network. Then *N* is tree-based if and only if $B_N$ is tree-based for every blob *B* in *N*.

### Proof

If *N* is tree-based, then it contains a support tree *T*. Since every cut-edge in *N* must be contained in a support tree, it follows that if *B* is a blob in *N*, then *T* must induce a spanning tree of $B_N$ that contains every vertex in $B_N$. Therefore, $B_N$ is tree-based.

Conversely, if $B_N$ is tree-based for every blob *B* in *N*, then by taking a support tree in $B_N$ for each blob *B* in *N*, we can clearly construct a spanning tree for *N* that contains all vertices in *N*. Therefore, *N* is tree-based.$\square$

Using the last result, we can immediately classify the tree-based networks having a single leaf.

### Observation 1

Suppose *N* is a network on $\{x\}$. Then *N* is tree-based if and only if $N=(\{x\},\emptyset )$.

We now look in more detail at the cut-edges of a tree-based network. A *split* of *X* is a bipartition of *X* into two non-empty sets. If we remove a cut-edge from a network, then in some cases the two resulting graphs will induce a split of *X*. We now show that if *N* is tree-based, then this is always the case.

### Lemma 2

If *N* is a tree-based network, then every cut-edge of *N* induces a split of *X*.

### Proof

If we have a cut-edge of *N* that does not induce a split of *X*, then it follows that there must be some blob *B* in *N* such that $B_N$ is a network with one leaf. But then $B_N$ is not tree-based by Observation 1. This is a contradiction by Proposition 1.$\square$

We call a network *N* *proper* if every cut-edge induces a split of *X*. By Lemma 2, all tree-based networks are proper.

Interestingly, using Proposition 1, we are able to now show that certain low complexity proper networks are always tree-based. We first make a useful observation.

### Lemma 3

Let *N* be a network on *X* with $|X|\ge 2$. For any $x\in X$ let $N-x$ denote the network obtained from *N* by deleting *x* and its incident edge, and suppressing the resulting degree 2 vertex. If $N-x$ is tree-based, then so is *N*.

### Proof

Suppose $x\in X$ and *T* is a support tree for $N-x$. Let $v\in V(N)$ denote the vertex adjacent with *x* that was suppressed in the construction of $N-x$ and let $e\in E(N-x)$ denote the resulting edge in $N-x$. If $e\in E(T)$, then we can obtain a support tree for *N* by subdividing *e* with a new vertex *w* and adding the edge $\{w,x\}$ to *T*. If $e\not \in E(T)$, then, since *T* is a support tree for $N-x$, *T* must contain both vertices in *e*, say $v_1$ and $v_2$. Therefore, we can obtain a support tree for *N* by adding a new vertex *w* and the edges $\{v_1,w\}$ and $\{w,x\}$ (or indeed $\{v_2,w\}$ and $\{w,x\}$) to *T*.

$$\square $$

Suppose *N* is a network on *X* and $k\ge 0$ is an integer. Then *N* is called a *level-k network* if at most *k* edges have to be removed from each blob of *N* to obtain a tree. For example, the network in Fig. 1 is a level-2 network.

### Theorem 1

All proper level-4 networks are tree-based. Moreover, networks of level greater than 4 need not be tree-based.

### Proof

First observe that by definition of level-*k* network, the main claim applies to level-1, 2, 3, and 4 networks.

Note that since *N* is a proper network on *X*, it must contain at least two leaves. Also note that the theorem is straight-forward to check in case *N* is level-0 or level-1.

**Fig. 2:**
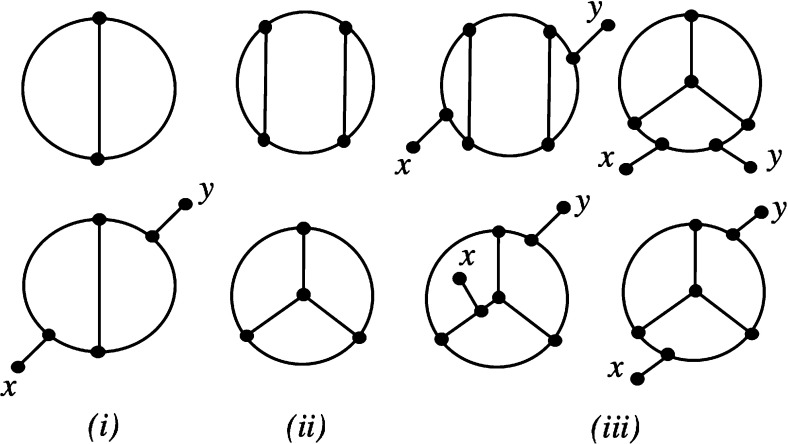
Diagram for proving that simple level-2 and level-3 networks are tree-based used in the proof of Theorem 1

In case *N* has level $2 \le k \le 4$, since *N* is proper, by Proposition 1 it suffices to prove that every simple, level-4 network with two leaves is tree-based. This is because we can decompose *N* into a collection of simple networks $B_N$ (one for each blob *B* of *N*) each having at least 2 leaves, and if each of these simple networks is tree-based, then so is *N*. Moreover, for each of these simple networks $B_N$, if we remove all but 2 leaves from $B_N$ and obtain a tree-based network, then it is straight-forward to see using Lemma 3 that $B_N$ must have been tree-based.

Now, to see that any simple, level-4 network with two leaves *x* and *y* is tree-based, we begin with the case $k=2$. It is clear that any simple level-2 network on some set *Y* can be obtained by inserting pendant edges in the multigraph at the top of Fig. 2(i) and labelling the leaves by the elements of *Y* (see, e.g. van Iersel and Moulton 2017, Figure 4). It is now straight-forward to check that, up to isomorphism, the only possible simple level-2 network on $\{x,y\}$ is isomorphic to the one at the bottom of Fig. 2(i). Clearly, this network is tree-based.

We now consider the case $k=3$. As before, it is known that any simple level-3 network on some finite set *Y* can be obtained by inserting a pendant edge into one of the multigraphs in Fig. 2(ii) and labelling the leaves by the elements of *Y* (van Iersel and Moulton 2017, Figure 4). It is now straight-forward to check that the only possible simple level-3 networks on $\{x,y\}$ are isomorphic to one of the networks in Fig. 2(iii), and that each of these is tree-based.

**Fig. 3:**
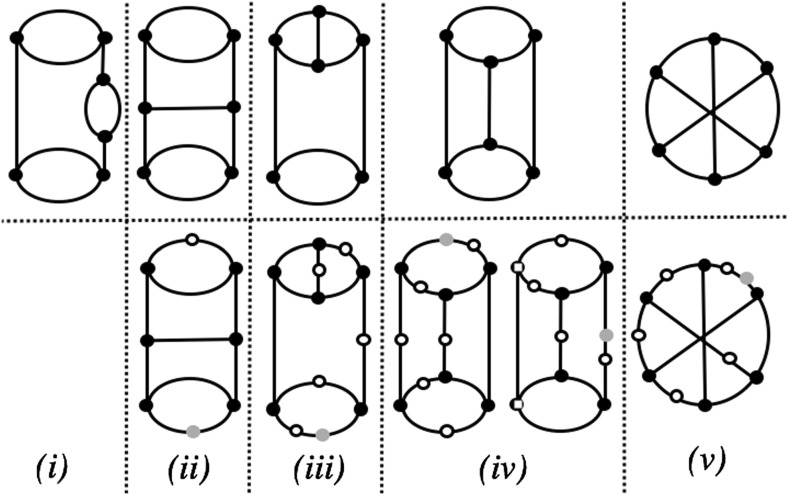
Diagram for proving that simple level-4 networks are tree-based used in the proof of Theorem 1. In the bottom row, each grey vertex corresponds to inserting a pendant edge labelled by *x*, and each circle vertex corresponds to inserting a pendant edge labelled by *y*, so that a network on $\{x,y\}$ is produced [so, for example, there are 5 possible networks associated to the diagram in the bottom row of column (iii)]

We conclude with the case $k=4$. We use the fact that any simple level-4 network with leaf set *Y* can be obtained by inserting pendant edges in one of the five multigraphs in the top row of Fig. 3 and labelling the leaves by the elements of Y (see, e.g. van Iersel and Moulton 2017, Figure 4). Using this fact, it is now straight-forward to check that, up to isomorphism, any simple level-4 network on $\{x, y\}$ is isomorphic to one of the networks on $\{x, y\}$ that can be generated from the bottom row of Fig. 3 as described in the figure’s caption [note that we can exclude the case (i) in the bottom row as it is not possible for this to be made into a simple network with leaf set $\{x, y\}$]. It is now straight-forward to check that each of these networks on $\{x, y\}$ is tree-based, which concludes the case $k=4$.

**Fig. 4:**
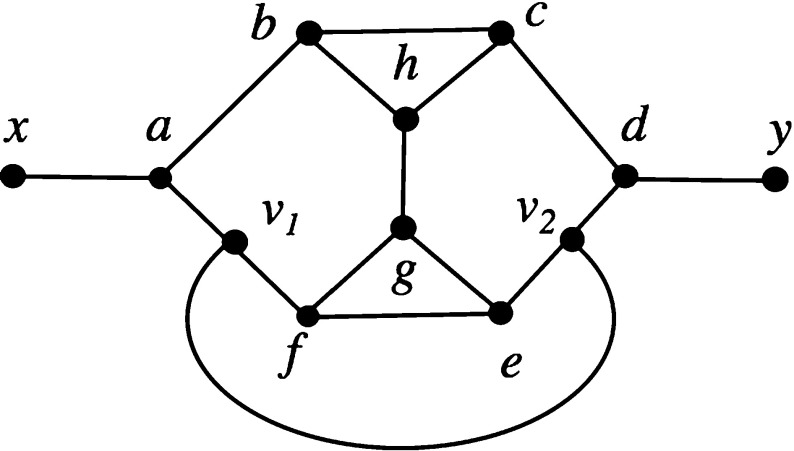
A level-5 network on $\{x,y\}$ that is not tree-based (Steel). The labels of the interior vertices are included for proof purposes

We now prove the last statement of the theorem. An example of a level-5 network is presented in Fig. 4. This network can be seen to be not tree-based as follows.

If it were tree-based, then there would be a path from *x* to *y* passing through every vertex exactly once. If the path began *x*, *a*, *b*, or ended *c*, *d*, *y*, then it could not pass through $v_1$ or $v_2$ without going through some vertex twice. Therefore, such a path begins $x,a,v_1$ and ends $v_2,d,y$. The edge $\{v_1,v_2\}$ cannot be included in such a path, because that completes the path without passing through all vertices, so the path actually must begin $x,a,v_1,f$ and end $e,v_2,d,y$. At this point the path cannot include vertices *b*, *c*, *h* without passing through *g* twice: a contradiction.

This example demonstrates that not all level-5 networks are tree-based, and since by definition of level, this network is also level-*k* for any $k\ge 5$, the result follows. $\square$

### Remark 1

The level-5 example used in the proof of Theorem 1 can be used to show that it is possible to construct networks that are *strictly* level-*k* [in that they are level-*k* and not level-$(k-1)$] and that are not tree-based. Take a network *N* of level-$k\ge 5$ and choose a pendant edge $\{x,y\}$ in *N*. Replace this edge by the level-5 network shown in Fig. 4. The resulting network has unchanged level and is not tree-based.

## Recognizing Tree-Based Networks

In this section, we consider the complexity of the computational problem of deciding whether or not a given network *N* is tree-based.

**Fig. 5:**
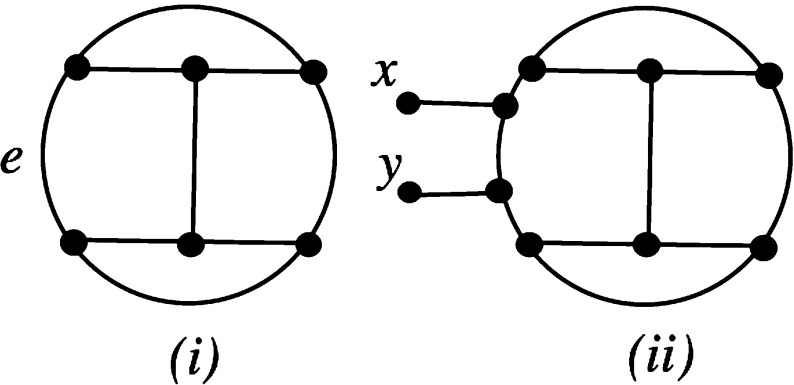
(i) Cubic graph *C* with edge *e* indicated. (ii) The network $C_e(x,y)$

We begin with a useful result. Suppose that *C* is a cubic graph. Pick some edge *e* in *C*. Introduce two pendant edges into *e* containing the new degree 1 vertices *x* and *y*. This new graph $C_e(x,y)$ is a network on $\{x,y\}$. We illustrate this construction in Fig. 5. The following observation concerning this construction is straight-forward to check.

### Lemma 4

Suppose that *C* is a cubic graph. The following statements are equivalent:

(i) *C* is Hamiltonian.
(ii) There is some edge *e* in *C* such that the network $C_e(x,y)$ is tree-based.
(iii) There is some edge *e* in *C* such that the network $C_e(x,y)$ has a support tree consisting of a path with end vertices *x* and *y*.

**Fig. 6:**
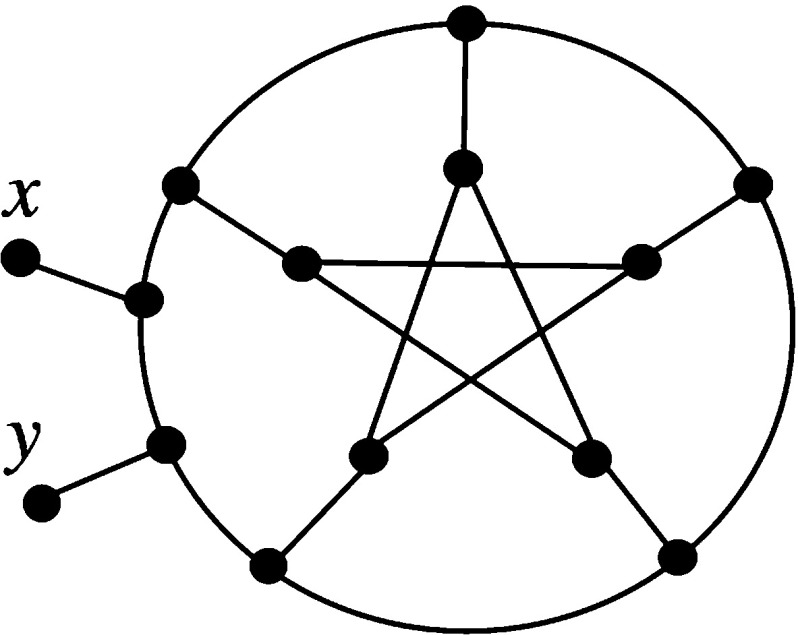
A simple level-6 network that is not tree-based. Removing the two pendant edges labelled with *x* and *y* and their vertices results in the Petersen graph

Note that using Lemma 4, it immediately follows that the network in Fig. 6 is not tree-based, since if *P* is the Petersen graph (which is not Hamiltonian), then this network is of the form $P_e(x,y)$ for some edge *e* of *P*.

We now use the last lemma to prove two NP-completeness results. In our proofs, we shall use the fact that the following problem is NP-complete (Garey et al. 1976):

*PC3C-Hamiltonian*

Instance: Planar, cubic, 3-connected graph *G*.

Question: Is *G* Hamiltonian?

We begin by showing that the following problem is NP-complete.

*Unrooted tree-based*

Instance: Network *N* on *X*.

Question: Is *N* tree-based?

### Theorem 2

The problem *Unrooted tree-based* is NP-complete.

### Proof

First note that *Unrooted tree-based* is in NP since we can check in polynomial time if a given tree in *N* is a spanning tree for *N* with leaf set *X*.

To complete the proof, we show that there is a polynomial-time reduction from *PC3C-Hamiltonian* to *Unrooted tree-based*.

Let *C* be a planar, cubic, 3-connected graph. By Lemma 4(ii) it follows that *C* is Hamiltonian if and only if the network $C_e(x,y)$ on the set $\{x,y\}$ is tree-based for some edge *e* in *C*. Since the number of edges in *C* is equal to 3|*V*(*C*)| / 2, it follows that there is a polynomial-time reduction from *PC3C-Hamiltonian* to *Unrooted tree-based* (just check if $C_e(x,y)$ is tree-based for each edge *e* in *C*; if the answer is no for every *e*, then *C* is not Hamiltonian; otherwise, *C* is Hamiltonian).$\square$

Interestingly, the analogous decision problem to *Unrooted tree-based* for rooted phylogenetic networks can be decided in polynomial time (Francis and Steel 2015).

We now prove that a related decision problem is NP-complete. We say that a phylogenetic tree *T* on *X* is *displayed* by a network *N* on *X* if *T* can be obtained from a subtree $T'$ of *N* by suppressing all degree 2 vertices in $T'$ (van Iersel et al. 2017). In addition, we say that *T is a base-tree of N* or *N is based on T* if *T* can be obtained in this way from a support tree $T'$ of *N*. Note that a phylogenetic tree may be displayed by a network but need not be a support tree for the network. For example, the phylogenetic tree consisting of an edge with leaves *x*, *y* is displayed by the network in Fig. 5 (e.g. consider the path of length 3 in the network between *x* and *y*), but it is not a support tree for that network.

We now consider the following decision problem.

*Unrooted base-tree containment*

Instance: Network *N* on *X* and a phylogenetic tree *T* on *X*.

Question: Is *N* based on *T*?

Note that the analogous version of this decision problem for rooted phylogenetic networks is NP-complete (Anaya et al. 2016). We now show that this is also the case for networks.

### Theorem 3

The problem *Unrooted base-tree containment* is NP-complete.

### Proof

First note that *Unrooted base-tree containment* is in NP since we can check in polynomial time if a subtree $T'$ of *N* is a support tree of *N*, and that a given phylogenetic tree *T* can be obtained from $T'$ by suppressing all degree 2 vertices in $T'$.

To complete the proof, we show that there is a polynomial-time reduction from *PC3C-Hamiltonian* to *Unrooted base-tree containment*.

Let *C* be a planar, cubic, 3-connected graph. By Lemma 4(iii) it follows that *C* is Hamiltonian if and only if there is some edge *e* in *C* such that the network $C_e(x,y)$ has a support tree consisting of a path with end vertices *x* and *y* which, in turn, holds if and only if the phylogenetic tree *T* consisting of a single edge and leaf set $X=\{x,y\}$ is a base-tree for the network $C_e(x,y)$ for some edge *e* in *C*. Since the number of edges in *C* is equal to 3|*V*(*C*)| / 2, it follows that there is a polynomial-time reduction from *PC3C-Hamiltonian* to *Unrooted base-tree containment* (just check if the single-edged phylogenetic tree *T* on $\{x,y\}$ is a base-tree for the network $C_e(x,y)$ for each edge *e* in *C*; if the answer is no for every *e*, then *C* is not Hamiltonian; otherwise, *C* is Hamiltonian).$\square$

Note that it is also NP-complete to decide whether or not a network *N* displays a phylogenetic tree *T* (van Iersel et al. 2017).

## Universal Tree-Based Networks

In this section, we shall show that there are networks on *X* which can have *every* phylogenetic tree on *X* as a base-tree. To prove this, we will relate networks with rooted phylogenetic networks, which we now formally define.

A *rooted* phylogenetic network *M* (on *X*) is a directed acyclic graph with a single root (vertex with indegree 0 and outdegree 2), leaf set *X* (vertices with indegree 1 and outdegree 0), and all vertices except the root having degree 1 or 3. If *M* is a tree, then it is called a *rooted* phylogenetic tree on *X*. A rooted phylogenetic network *M* is called *tree-based* if it contains a directed spanning tree (an “arborescence”) *T* such that the leaf set of *T* is *X*. In that case, *T* is called a *support tree* for *M*.

In Hayamizu (2016) it is shown that for every *X* there exists a “universal” rooted phylogenetic network *M* on *X*, that is, *M* is a tree-based, rooted phylogenetic network and has every possible rooted phylogenetic tree on *X* as a base-tree. We shall use this result to show that there are also universal networks. First, we present a relationship between tree-based networks and rooted phylogenetic networks (cf. also Gambette et al. 2012, Section 3 for related results).

Given a network *N* on *X* with $|X|\ge 2$, a leaf $x \in X$, and some orientation *o* of the edges of *N*, we let $N_o^x$ denote the directed graph which results by removing *x* and its pendant edge from *N* with edges oriented according to *o*.

### Theorem 4

Suppose that *N* is a network on *X* and $x\in X$. Then *N* is tree-based if and only if there exists some orientation *o* of the edges of *N* making $N_o^x$ a tree-based (rooted) network on $X-\{x\}$.

### Proof

Suppose *o* is an orientation of the edges of *N* such that $N_o^x$ is a tree-based, rooted network on $X-\{x\}$. Pick some base-tree *T* in $N_o^x$. Let $v_x$ denote the vertex in *N* that is adjacent with *x* and let $v_1\in V(N)$ denote one of the two other vertices in *N* adjacent with $v_x$. Then $v_1\in V(T)$. Let $T'$ be the tree obtained from *T* by first adding *x* to its leaf set, $v_x$ to its vertex set, and $\{v_x,x\}$ and $\{v_1,v_x\}$ to its edge set and then ignoring the directions of the edges of *T*. Since *T* is a spanning tree of $N_o^x$ with leaf set $X-\{x\}$, we clearly have that $T'$ is a spanning tree for *N* with leaf set *X*. Thus, *N* is tree-based.

Conversely, suppose that *N* is tree-based. Pick some support tree *T* in *N* and orient all edges in *T* away from *x*. Let $x,v_1,v_2,\dots ,v_m$ be some topological ordering of the vertices in *T* (i. e.  an ordering that is consistent with the partial ordering induced by *T*). For each vertex *v* in *T* that is the end vertex of some edge in *N* not in *T*, starting with a vertex that comes earliest in the ordering, direct the edge away from *v*, and if such an edge is encountered that has already been directed, then ignore this. This choice *o* of orientations of the edges of *N* implies that $N_o^x$ is a rooted phylogenetic network on $X-\{x\}$ (since it has no directed cycles) with support tree $T_{o'}^x$ where $o'$ is the orientation of the edges induced by *o*.$\square$

### Corollary 1

There exists a tree-based network *N* on *X* such that every phylogenetic tree *T* on *X* is a base-tree for *N*.

### Proof

The cases $|X|=1$ and $|X|=2$ are obvious. Assume $|X|\ge 3$. Let $x \in X$ and set $Y =X -\{x\}$. Let *M* be a universal rooted network on *Y* (see Hayamizu 2016 for details). Let $\rho$ denote the root of *M*. Let *N* be the network on *X* obtained by adding *x* to the leaf set of *M*, a new vertex *r* to the vertex set of *N*, new edges $\{r,x\}$ and $\{r,\rho \}$ to *M*, and ignoring the orientations of all edges of *M*. Clearly, *N* is a network on *X* and *M* and $N_o^x$ are isomorphic where *o* is the orientation of the edges of *M*. By Theorem 4, *N* is tree-based. Moreover, if *T* is any rooted phylogenetic tree on *Y*, then *T* is a base-tree for *M* because *M* is a universal network on *Y*. Hence, the tree $T_x$ obtained by adjoining the element *x* as a leaf to the root of *T* is a phylogenetic tree on *X* and ignoring its edge orientations renders it a base-tree for *N*. But it is straight-forward to check that the set

$$\begin{aligned} \{ T_x \,:\, x\in X \text{ and } T \text{ a } \text{ rooted } \text{ phylogenetic } \text{ tree } \text{ on } X-\{x\} \} \end{aligned}$$

is equal to the set of phylogenetic trees on *X*. The corollary follows immediately.$\square$

## Fully Tree-Based Networks

In Semple (2016) a characterization of rooted phylogenetic networks in which every embedded phylogenetic tree with the same leaf set is a base-tree is given (these are precisely the “tree-child” networks). In our last result, we will characterize networks that have an analogous property.

Note that a network *N* on *X* always contains a subtree with the same leaf set as *N*. For example, if we fix some $x \in X$ and let $p_{xy}$ be some path in *N* for all $y \in X -\{x\}$, then the tree obtained by removing (if necessary) a minimum number of edges from the union of the paths $p_{xy}$ over all $y \in X-\{x\}$ is a subtree of *N* with leaf set *X*.

We call a network *N* on *X* *fully tree-based* if every subtree of *N* with leaf set *X* is a support tree for *N*. Note that by the previous remark, any fully tree-based network is tree-based.

### Lemma 5

Suppose that *N* is a simple, tree-based network and that *T* is a base-tree for *N*. If $e=\{v_1,v\},e'=\{v,v_2\}$ are incident edges in *T* such that neither *e* nor $e'$ are pendant edges of *T*, and $T_e$ and $T_{e'}$ are the trees which are obtained by deleting *e* and $e'$, respectively, that do not contain *v*, then there must exist some edge $e''$ in *N* which has one vertex in $T_e$ and the other in $T_{e'}$.

### Proof

Suppose that there is no edge $e''$ with the stated properties. Then there exists only one path in *N* between $v_1$ and $v_2$. But this contradicts the fact that *N* is simple, and therefore the graph *N* with all pendant edges removed is 2-connected.$\square$

### Lemma 6

Suppose that *N* is a simple, level-*k* network, $k\ge 1$, on *X*, $|X| \ge 2$. Then *N* contains a vertex which is not contained in a pendant edge of *N* if and only if $k \ne 1$.

### Proof

Since *N* is simple, $|V(N)|=2(|X|-1+k)$ (cf., e.g. Huber et al. 2016). Now, let *q* be the number of vertices in *N* which are not contained in any pendant edge of *N*. Then clearly

$$\begin{aligned} |V(N)|= 2|X|+ q. \end{aligned}$$

Therefore, $q=2k-2$. The lemma now follows immediately.$\square$

We now characterize fully tree-based networks. Note that these are significantly less complex than the tree-child networks mentioned above.

### Theorem 5

Suppose that *N* is a network on *X*. Then *N* is fully tree-based if and only if *N* is a level-1 network.

### Proof

The statement is clearly true if $|X|=1$. So we assume from now on that $|X|\ge 2$.

By Proposition 1, it suffices to assume that *N* is simple.

If *N* is a simple, level-1 network, then it is straight-forward to check that it is fully tree-based.

Conversely, suppose for contradiction that *N* is a simple network on *X* which is not level-1, and that *N* is fully tree-based. Let *T* be a support tree for *N*.

Suppose that *v* is a vertex in *T* that is not contained in some pendant edge of *N*. Note that such a vertex exists by Lemma 6 since *N* is not level-1.

If the degree of *v* in *T* is 2, then let $e=\{v_1,v\},e'=\{v,v_2\}$ denote its incident edges neither of which can be a pendant edge in *N*. Then, by Lemma 5, we can remove edges $e,e'$ from *T* and add in an edge $e'' \in E(N)$ in between a vertex of $T_e$ and a vertex of $T_{e'}$ where $T_e$ and $T_{e'}$ are as in the proof of that lemma. Since the degree of *v* in *T* is 2, the resulting tree $T'$ has leaf set *X*. Moreover $T'$ does not contain the vertex *v*. But this contradicts the fact that *N* is fully tree-based.

If the degree of *v* in *T* is 3, then let $e=\{v_1,v\},e'=\{v,v_2\}$ be two edges incident with *v*. Then by Lemma 5, there is an edge $e''$ between a vertex of $T_e$ and a vertex of $T_{e'}$. Now, if we remove *e* from *T* and add in edge $e''$, we obtain a new tree $T'$ that is a support tree for *N* and which contains a vertex (namely *v*) with degree 2, such that neither of the edges in $T'$ incident with *v* is a pendant edge of $T'$. But this is impossible by the argument presented above.$\square$

## Final Remarks

We have proven various results concerning tree-based networks and shown that they have somewhat different properties as compared with rooted tree-based networks.

The structure of rooted tree-based networks is very well understood see, e.g. Francis et al. (2018) and Zhang (2016). It would be interesting to know if related structural results can be proven for tree-based networks. In addition, results have recently appeared concerning the structure of non-binary rooted, tree-based networks (Jetten and van Iersel 2016) (networks in which internal vertices do not necessarily have degree 3). It would therefore also be of interest to consider properties of non-binary tree-based networks.

## Footnotes

1 Recall, a *spanning tree* of a graph *G* is a connected subgraph of *G* containing all vertices of *G* and a minimal subset of the edges.
